# Anomalous Cerebellar Anatomy in Chinese Children with Dyslexia

**DOI:** 10.3389/fpsyg.2016.00324

**Published:** 2016-03-18

**Authors:** Ying-Hui Yang, Yang Yang, Bao-Guo Chen, Yi-Wei Zhang, Hong-Yan Bi

**Affiliations:** ^1^Key Laboratory of Behavioral Science, Institute of Psychology, Chinese Academy of SciencesBeijing, China; ^2^The University of Chinese Academy of SciencesBeijing, China; ^3^Department of Linguistics, University of Hong KongHong Kong, China; ^4^State Key Laboratory of Brain and Cognitive Sciences, University of Hong KongHong Kong, China; ^5^School of Psychology, Beijing Normal UniversityBeijing, China; ^6^School of Labor and Human Resources, Renmin University of ChinaBeijing, China

**Keywords:** developmental dyslexia, Chinese, voxel-based morphometry, left cerebellum, gray matter

## Abstract

The cerebellar deficit hypothesis for developmental dyslexia claims that cerebellar dysfunction causes the failures in the acquisition of visuomotor skills and automatic reading and writing skills. In people with dyslexia in the alphabetic languages, the abnormal activation and structure of the right or bilateral cerebellar lobes have been identified. Using a typical implicit motor learning task, however, one neuroimaging study demonstrated the left cerebellar dysfunction in Chinese children with dyslexia. In the present study, using voxel-based morphometry, we found decreased gray matter volume in the left cerebellum in Chinese children with dyslexia relative to age-matched controls. The positive correlation between reading performance and regional gray matter volume suggests that the abnormal structure in the left cerebellum is responsible for reading disability in Chinese children with dyslexia.

## Introduction

Developmental dyslexia (DD) is characterized by an unexpected difficulty in reading, which is not explained by intellectual impairment, sensory deficits, lack of adequate schooling opportunities, or neurological illness. It is a common developmental disorder affecting 5–18% of school-aged children (Snowling, 2000). The phonological deficit theory is widely accepted in alphabetic languages, suggesting that dyslexics have specific sound manipulation impairments, which affects their auditory memory, word recall, and sound association skills when processing speech (Shaywitz et al., 1998; Ramus, 2001; Hoeft et al., 2007a,b; Ramus and Ahissar, 2012; Boets et al., 2013; Ramus et al., 2013). However, some researchers argue that deficits at the linguistic level may be an external manifestation of DD, and linguistic deficits can be traced back to a more general perceptual deficit, namely, a dysfunction of magnocellular in sensory pathways. The magnocellular deficit theory asserts that the reading problems derive from impaired sensory processing, caused by abnormal visual, auditory or tactile modalities (Stein and Walsh, 1997). Meanwhile, the cerebellar hypothesis for DD indicates that the cerebellar disorder leads to motor control, or automatization difficulties that would subsequently cause reading and writing problems (Nicolson et al., 2001). Furthermore, as the cerebellum receives massive input from various magnocellular systems, it is also proposed that the cerebellar deficit should be unified under the generally magnocellular theory of dyslexia (Stein, 2001). The cerebellar deficit hypothesis has been supported extensively by behavioral, neuroanatomical and neuropsychological studies (Nicolson et al., 1999; Brown et al., 2001; Rae et al., 2002; Stoodley et al., 2008). For example, compared with typically developing readers, children, and adults with dyslexia performed poorer in several tasks related to cerebellar function, such as time estimation (Wolff et al., 1990), automatic balance (Brookes et al., 2010), speed processing (Nicolson et al., 1995), and implicit motor learning (Stoodley et al., 2008). Using positron emission tomography (PET), Nicolson et al. (1999) directly examined the cerebellar function in learning novel sequences and executing prelearned sequences in adults with dyslexia. It was found that relative to control group, dyslexics exhibited reduced activation in the right cerebellar cortex during both learning novel sequences and executing prelearned sequences. Menghini et al. (2006) found that in alphabetic languages, adults with dyslexia showed increased activation in the right cerebellum than the normal control group. Adults with dyslexia showed less activation in the left cerebellum than the control group during word and pseudoword reading (McCrory et al., 2000). In a meta-analysis, Linkersdörfer et al. (2012) identified that the structural abnormalities were related to functional abnormalities in bilateral cerebellar lobes in dyslexics. Anatomical cerebellar abnormality in dyslexia was supported by the evidence showing the absence of cerebellar asymmetry in dyslexic children (Rae et al., 2002). Structural magnetic resonance imaging (MRI) studies of dyslexia using voxel-based morphometry (VBM) have revealed regional gray matter reductions in the right cerebellum (Brown et al., 2001; Pernet et al., 2009), or in the bilateral cerebellum (Eckert et al., 2003, 2005; Brambati et al., 2004; Kronbichler et al., 2008). However, Richlan et al. (2013) did not find significant cerebellar volume reduction in dyslexics. In general, most of the above studies showed abnormal cerebellar activation and structure in right or lateral lobules in dyslexia of alphabetic languages.

But different from alphabetic languages, Chinese is a logography without clear grapheme-phoneme rules, and Chinese characters are square-shaped with more visually complicated structures. These differences may lead to different neural basis of Chinese character processing (Bolger et al., 2005; Tan et al., 2005; Wu et al., 2012). For example, several neuroimaging studies of normal readers have shown activation in the left middle frontal gyrus (MFG; BA 9) during Chinese reading, which was thought to be specialized for orthography-to-phonology transformation in Chinese processing (Tan et al., 2005; Siok et al., 2008), while in alphabetic-language reading, the left posterior temporal lobe was recruited to perform the conversion of written symbols (letters) into phonological units of speech (phonemes) (Booth et al., 2002). The right parietal and inferior occipital cortices were thought to be engaged in visuospatial analysis in processing Chinese characters (Tan et al., 2001; Chen et al., 2002), while the right superior frontal gyrus, right parietal regions and bilateral cuneus were known to be critical for visuospatial processes in processing alphabetic words (Haxby et al., 1995; Lepage et al., 2000). Thus, there is a discrepancy of the neural basis between Chinese and alphabetic languages processing.

In neuroimaging studies of dyslexia, Siok et al. (2004, 2008) have found that, compared with typically developing reading, dyslexic reading in Chinese was characterized by reduced activation in the left MFG during homophonic (Siok et al., 2004) or rhyming (Siok et al., 2008) judgment. While some studies found that the activation in the left temporoparietal and occipitotemporal regions was abnormally decreased during alphabetic-language reading in dyslexia (Horwitz et al., 1998; Johansson, 2006; Schlaggar and McCandliss, 2007). Hu et al. (2010) found a similar pattern of brain activity in semantic decision tasks in Chinese and English people with dyslexia, both of them showing reduced activation in the left angular gyrus, left middle frontal cortex, and left occipitotemporal regions relative to normal readers even though Chinese and English normal readers displayed distinct activation in the brain. Therefore, it is not clear whether the neural basis of deficits in DD varies across languages.

As for research in cerebellar deficits of Chinese dyslexia, one behavioral study found that Chinese dyslexia had problems in implicit motor learning when they responded with their left hands, whereas this problem disappeared when using their right hands. In contrast, age-matched children showed significant implicit motor learning when responded with either hand (Yang and Hong-Yan, 2011). The observation of the left-hand response deficits during implicit motor learning in Chinese dyslexia led the researchers to speculate that Chinese dyslexia is likely to be associated with left cerebellar dysfunction, which may be different from the previous studies of cerebellar deficits in alphabetic-language dyslexics (Nicolson et al., 1999; Menghini et al., 2006). Yang et al. (2013) have performed a functional magnetic resonance imaging (fMRI) study to examine cerebellar function in an implicit motor learning task in children with and without dyslexia. The results indicated that Chinese children with dyslexia had significantly higher activity in the left cerebellum compared with age-matched normal children (Yang et al., 2013). Thus, these findings suggested different cerebellar deficits in Chinese and alphabetic dyslexia. As discussed previously (Linkersdörfer et al., 2012), functional deficits usually come along with structural defects. Therefore, this study aimed to determine whether there were structural abnormalities in the left cerebellum in Chinese dyslexia.

## Materials and Methods

### Participants

Nine dyslexic children (3 boys, mean age = 12.6 years, *SD* = 0.6) and 14 normal control group (6 boys, mean age = 12.3 years, *SD* = 1.0) took part in the study. The children were recruited from ordinary primary schools in Beijing. Two tests that were widely used for screening Mandarin-speaking Chinese children with dyslexia were adopted: the Raven Standard Progressive Matrices (Raven et al., 1996), and the Character Recognition Test and Assessment Scale (Wang and Tao, 1993). The vocabulary test used in the present study is a standardized vocabulary test for screening DD in Mainland China. In this test, the children were required to write a compound word using a given Chinese character. Each correctly used character was given one point. It includes 210 characters which are divided into 10 sub-groups based on their reading difficulty, which corresponds with the standard difficulty coefficient. The score for each sub-group was calculated by multiplying the total points by the corresponding coefficient of reading difficulty. The final score was measured by adding the total score of 10 sub-groups’ and the constant which was the number of characters almost all children in the same grade could recognize. It is obvious that, raw scores of the test are measured based on the standard difficulty coefficient. And many previous studies with Chinese dyslexia used the raw scores of this vocabulary test to select dyslexia children (Shu et al., 2006; Meng et al., 2007; Wang et al., 2010; Yang and Hong-Yan, 2011; Liu et al., 2012, 2013; Yang et al., 2013; Qian and Bi, 2014; Zhao et al., 2014, 2015). Besides, in this current study, the inclusionary criterion for dyslexics was their written score at least 1.5 standard deviation (*SD*) below the average score of all participants, not a fixed score. In addition, a rapid digit naming task was administered, in which five digits (2, 7, 4, 9, and 6) were presented in random order on a 6 × 5 column grid. All children were asked to read the 30 Arabic digits twice as quickly and accurately as possible. The reading time was recorded. This test was adopted to measure children’s rapid automatized naming ability (Zhao et al., 2014). The inclusion criteria for selecting DD were as follows: (1) their reading scores were at least 1.5 *SD* below the average score of age-matched children; and (2) the children had an IQ score higher than 85 in the Raven test. Detailed information of participants was presented in **Table 1**. None of the children suffered from attention deficit/hyperactivity disorder (ADHD) according to the scores of the Chinese Classification of Mental Disorder 3 (CCMD-3). All children had no history of sensory deficits or neurological or psychiatric illness. All participants were right-handed based on the Handedness Inventory (Department of Neurology, Beijing Medical University Hospital). The study was approved by the Ethics Committee of the Institute of Psychology, Chinese Academy of Sciences, and written informed consent was obtained from all participants’ guardians.

**Table 1:** Information concerning the dyslexia and control groups.

	Dyslexic readers (*n* = 9)	Normal readers (*n* = 14)	*p*	η^2^
	Mean (*SD*)	Mean (*SD*)		
Age (years)	12.6 (0.61)	12.3 (1.0)	>0.05	0.15
Vocabulary (standard score)	2487.39 (149.94)	3101.52 (123.36)	<0.001	0.794
Raven (standard score)	110.35 (15.52)	114.26 (10.35)	>0.05	0.074
Time of digit naming (seconds)	10.63 (1.03)	9.34 (1.01)	<0.01	0.294

### MRI Acquisition

The MRI data were obtained on a 3 Tesla Siemens MAGNETOM Trio scanner (Siemens, Erlangen, Germany) with a standard head coil. A T1-weighted gradient-echo planar imaging (EPI) sequence was used for each subject’s high-resolution whole-brain images, with the repetition time = 25 ms, echo time = 30 ms, field of view = 25 mm, matrix size = 256 × 256, voxel size = 1 mm × 1 mm × 1 mm, and 128 non-contiguous (gapped) slices of 4-mm thickness.

### Image Processing and Analysis

Image analysis was performed using SPM8 software^1^ (Statistical Parametric Mapping) in MATLAB 7.8 (R2009a) (Math Works, Natick, MA, USA). T1-weighted images were analyzed using the VBM8 toolbox^2^. Spatial normalization was achieved by registering each image to the standard T1 template implanted in SPM8, based on the Montreal Neurological Institute (MNI) stereotactic space. In the present analysis, the first step in spatially normalizing each image involved matching the image by estimating the optimum parameter affine transformation, and then estimated the coefficients of the basic functions to minimize the residual squared difference between the image and the template by using the non-linear registration. The spatially normalized images were partitioned into gray matter, white matter and cerebral spinal fluid with a resampling at 1 mm × 1 mm × 1 mm resolution, using a modified mixture cluster analysis technique. The segmented images were then modulated (to correct for local expansion or contraction) by dividing with the Jacobian of the warp field. The modulated segmented gray matter images were smoothed with an isotropic Gaussian kernel with a full width at half maximum of 8 mm. The actual volumes of the entire normalized, segmented, and restored segmented images were determined by adding the voxel volumes (1 mm × 1 mm × 1 mm), and multiplying by each voxel value. Intracranial volume was determined by adding the gray matter, white matter, and cerebrospinal fluid space volumes.

No participants from the two groups were excluded because of movement artifacts or incomplete brain scans. We conducted whole-brain gray matter volume analysis using SPM8. Statistical parametric maps of whole-brain VBM analyses were displayed on a template brain. Group differences in the whole-brain gray matter were assessed with the two-sample *t* statistic within SPM software, with total intracranial volume as a covariant; the corrected statistical threshold was set at *p* < 0.001, corrected by AlphaSim correction, extent threshold *k* = 111 voxels. Coordinate points of regions of interest (ROI) were generated and labeled based on regions showing significant group differences in gray matter volume in the above VBM analysis. ROI analysis was used to confirm the statistical parametric map results and to perform correlational analyses by extracting mean gray matter volumes from all participants. Average gray matter volumes of these ROIs for each individual were extracted using the MarsBaR toolbox^3^ with 6 mm radius sphere centered at the peak of the group difference.

Structural covariance analysis was used to investigate whether there was significant covariance in gray matter volume among the left cerebellum and other brain regions showing group differences in volumes in the VBM analysis. This method has been used previously to examine gray matter correlations between regional volumes of circumscribed brain regions (Mechelli et al., 2005; Pernet et al., 2009; Liu et al., 2013). For structural covariance analysis, the threshold was set at *p* < 0.005 uncorrected, extent threshold *k* = 40 voxels. Total gray matter volume, age, and gender were included as covariates in the follow-up analyses for all participants to investigate whether the group differences in the structural co-variation remained significant after regressing out these factors of no interest.

## Results

### Comparison of Gray Matter Between Children With Dyslexia and Controls

**FIGURE 1:**
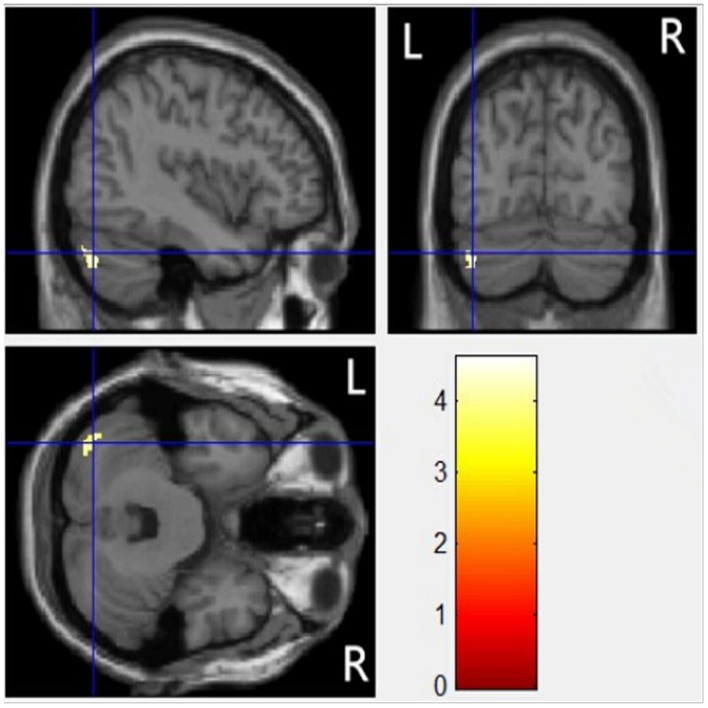
**Statistical parametric maps of whole-brain VBM analyses displayed on a template brain.** A region in the left posterior cerebellum exhibited significantly reduced gray matter volume in the dyslexic group compared with controls.

Voxel-based morphometry analysis showed that regional gray matter volume in the left cerebellar posterior lobe was significantly smaller in dyslexia than that in controls (MNI coordinates: –42/–73/–34; AlphaSim corrected, *t* = 4.61, cluster threshold *p* < 0.001, *k* = 111 voxels; as shown in **Figure 1**). There was no significant difference in total gray matter [*t* = –0.17, *p* = 0.86] and whole brain volume [*t* = 0.81, *p* = 0.42] between children with dyslexia and controls.

We reduced the statistical threshold to *p* < 0.005 uncorrected and extent threshold *k* = 40 voxels. VBM analysis revealed decreased gray matter volume in more widespread regions in children with dyslexia compared to controls, including the left superior temporal region (BA 38), left lateral orbitofrontal cortex (LOFC; BA 47), left MFG (BA 9), left postcentral gyrus (BA 41), and some right brain regions, such as the right cerebellum, right superior frontal gyrus (BA 6), and right fusiform (BA 37). Some regions showed an increase in gray matter in children with dyslexia compared with normal controls located in the right middle temporal gyrus (BA 21), right superior occipital gyrus (BA 18), and right precuneus (BA 7; as shown in **Table 2**).

**Table 2:** Gray matter volume comparisons.

Hemisphere lobe	Anatomical location	MNI coordinate			*Z*-score	Volume (voxels)
		*x*	*y*	*z*		
**Dyslexics < controls**						
R(cerebellum)	Cerebellum posterior	45	–63	–36	3.86	139
L(temporal)	Superior temporal gyrus	–40	10	–22	3.67	72
L(frontal)	Lateral orbitofrontal cortex	–49	16	3	3.66	53
L(frontal)	Middle frontal gyrus	–33	49	15	4.19	46
L(parietal)	Postcentral gyrus	–57	–21	14	4.31	42
R(frontal)	Superior frontal gyrus	25	12	60	4.81	52
R(occipital)	Fusiform gyrus	36	–67	–15	5.46	40
**Dyslexics > controls**						
R(temporal)	Middle temporal gyrus	42	9	–40	7.03	55
R(occipital)	Superior occipital gyrus	18	–87	22	4.18	50
R(parietal)	Precuneus	12	–52	42	6.39	45

### Structure-Behavior Correlation and Structural Covariance Analysis

**FIGURE 2:**
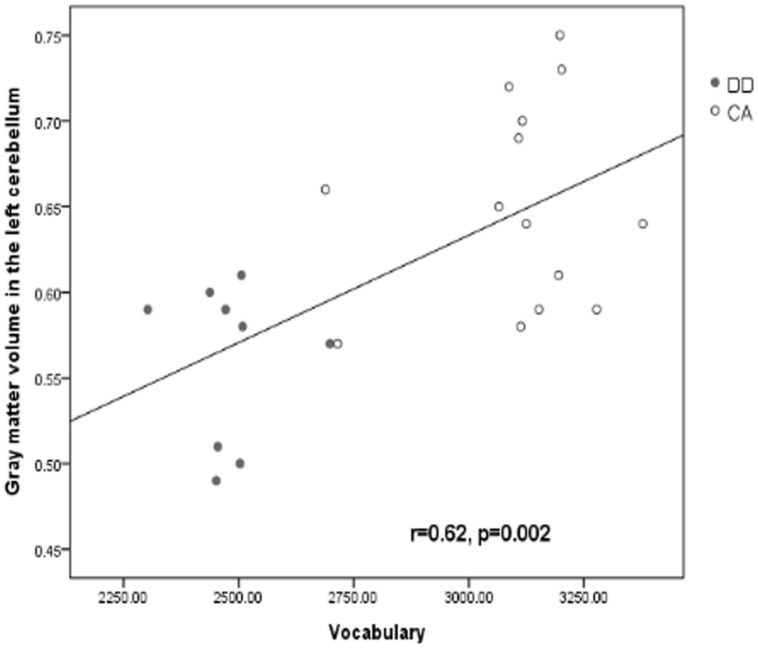
**Correlation between vocabulary score and gray matter volume in the left cerebellum.** As showed in the scatter plot, the black line represents the correlation line for all participants.

Gray matter volumes in the left cerebellum were correlated with reading scores (vocabulary) for all participants (*r* = 0.62, *p* = 0.002; as shown in **Figure 2**).

Gray matter correlations between the left cerebellum and other regions were also analyzed for all participants. The gray matter of the left cerebellum was significantly correlated with gray matter of several regions in the left hemisphere involving the left superior temporal gyrus (STG; *r* = 0.52, *p* = 0.010), the left LOFC (*r* = 0.48, *p* = 0.020), and the left postcentral cortex (*r* = 0.61, *p* = 0.002) and meanwhile was marginally correlated with the left MFG (*r* = 0.39, *p* = 0.062). Critically, the left cerebellum showed significant positive correlations with brain areas in the right hemisphere involving the right cerebellum (*r* = 0.69, *p* = 0.000) and right fusiform gyrus (*r* = 0.45, *p* = 0.031), and significant negative correlations with the right precuneus (*r* = –0.48, *p* = 0.020). These co-variations were still significant after regressing out the effect of total gray matter, age, and gender. In sum, children with less gray matter in the left cerebellar region tended to show decreased volumes in the right cerebellum, right fusiform, and increased volumes in the right precuneus.

## Discussion

The major finding of the present study was that children with dyslexia displayed a significantly reduced gray matter volume in the left cerebellum, and gray matter volume of the left cerebellum was positively correlated with vocabulary score. Volume of the left cerebellum was positively related to the volume of the right cerebellum, the right fusiform gyrus, and negatively associated with the volume of the right precuneus.

The remarkable finding was decreased gray matter volume of the left cerebellum in Chinese dyslexic children. It was proposed that structural abnormality came along with functional abnormality deficits (Linkersdörfer et al., 2012). Combined with Yang et al. (2013) previous fMRI study which presented that the left cerebellum showed significantly higher activation in Chinese DD during motor sequence learning, we could see that Chinese people with dyslexia indeed have both functional and anatomical deficits in the left cerebellum. What’s more, the cerebellum deficits of Chinese reading dyslexia in this study was found in the left side, different from the right cerebellar abnormality found in previous studies of alphabetic-language dyslexics (Nicolson et al., 1999; Brown et al., 2001; Menghini et al., 2006; Pernet et al., 2009). This might reflect a unique neural mechanism of Chinese processing.

Much research has shown that Chinese and alphabetic languages have different neural basis and networks. For example, in word-form processing, an additional right middle occipital gyrus was utilized to process the holistic visuospatial configuration of Chinese characters, while this region was not activated during alphabetic-language reading (Bolger et al., 2005; Tan et al., 2005; Cao et al., 2010). Besides, during phonological processing, the left MFG was associated with the phonological processes of character-syllable mapping in Chinese (Siok et al., 2008). While in alphabetic languages, many left brain regions such as the left inferior frontal gyrus (IFG), the left STG, and left inferior parietal lobule (IPL) were involved in phonological processing. The left IFG was thought to be associated with alphabetic word reading (Fiez et al., 1999; Cornelissen et al., 2009) as well as phoneme manipulation and phonological rehearsal before speech production (Fiez, 1997; Price, 2010). The left IPL was responsible for grapheme-phoneme conversion (Paulesu et al., 2000; Booth et al., 2003), and the left STG has been found to be associated with fine-grained phonological representation (Booth et al., 2003; Temple et al., 2003; Nakamura et al., 2006). In the current study, our major finding was that Chinese children with dyslexia exhibited the reduction of gray matter volume in the left cerebellum, while dyslexia of alphabetic languages seemingly usually presented the right cerebellum deficits (Brown et al., 2001; Pernet et al., 2009). So our finding provides a new evidence to demonstrate the different neural basis for different language writing systems.

The present results also showed the volume of left cerebellum was correlated with vocabulary scores, which may suggest the cerebellum is closely related to reading ability, supporting cerebellar deficit hypothesis (Nicolson et al., 2001). It has been reported that the cerebro-cerebellar circuitry regulates the higher order cognitive processing, such as language processing, executive control functions, and working memory (Murdoch, 2010). Clinical and anatomical studies have shown crossed reciprocal connections of the Crus I/II (posterior cerebellar lobe) with the dorsolateral prefrontal cortex (BA 9/46) (Petrides and Pandya, 1999; Middleton and Strick, 2001), and inferotemporal and posterior parietal cortices (BA 7) (Ramnani, 2006; Jissendi et al., 2008). The perspective on cerebellar involvement in language stems from the cerebro-cerebellar interactions in linguistic functions (Cotterill, 2001; Marien et al., 2001; Schlaggar and McCandliss, 2007). The reciprocal connections between the cerebellum and Broca’s language area (lateral temporal and inferior frontal cortices) have also been demonstrated by functional neuroimaging studies, indicating that the cerebellum may be engaged in modulating both language production and comprehension (Petersen et al., 1989; Gebhart et al., 2002; Hubrich-Ungureanu et al., 2002; Jansen et al., 2005; Murdoch and Whelan, 2007; Stoodley and Schmahmann, 2009). It was the left cerebellum volume correlated with reading abilities in this study in Chinese dyslexia. The left cerebellum should be connected with the right cerebral cortex (Schmahmann, 1996; Middleton and Strick, 2001; Salmi et al., 2010). In fact, numerous studies have shown greater involvement of the right hemisphere in Chinese language processing, such as the right ventral occipital cortex, right superior and right IPLs (Tan et al., 2000; Liu and Perfetti, 2003; Xi et al., 2010; Wu et al., 2012). Therefore, we conjecture the deficits in the left cerebellum of Chinese dyslexic children might affect language processing in Chinese DD via influencing the activation of the right language-related cerebral cortex which is essential for Chinese reading, through the cerebro-cerebellar network.

The results also showed that the volume of the left cerebellum was positively related with the volume of the right fusiform gyrus and negatively correlated with right precuneus. Previous Chinese studies have demonstrated that the right ventral occipital cortex showed greater activation in orthographic processing of Chinese characters (Tan et al., 2001), or visual spatial analysis of characters (Liu and Perfetti, 2003; Bolger et al., 2005). On the other hand, the right precuneus (BA 7) was reported to be involved in visuospatial processes and reinstatement of visual images associated with remembered words (Kjaer et al., 2001; Cavanna and Trimble, 2006). The right precuneus was also proved to be a part of the reciprocal neuroanatomical connections between the cerebellum and cerebral cortex (prefrontal, temporal and parietal cortices) (Buckner et al., 2011; Stoodley, 2012). Chinese character is more complex than alphabetic languages, which needs more visuospatial processing. The correlations between the left cerebellum and right fusiform gyrus and right precuneus might further suggest that the left cerebellum might play a role in the visual processing of Chinese character during reading through its connection with viusospatial processing regions. This finding might also lend some support to the cerebellum hypothesis (Nicolson et al., 2001). An alternative explanation about the negative correlation between the left cerebellum and right precuneus may be resolved by evidence from human (Chang et al., 2005) and animal (Rosen et al., 2000) studies indicating that connectivity disorders between neighboring brain regions may lead to atrophic changes. These changes might include decreased gray matter. The negative correlation of increased gray matter in the right precuneus could be a result of experience-dependent structural remodeling of cortical circuits underlying the acquisition of skills (May, 2011).

What’s more, in the current study, some regions also showed gray matter volume reductions in dyslexic children when the statistical threshold reduced to *p* < 0.005, such as the left MFG and the LOFC. Based on the previous findings, the left MFG played a particularly important role in Chinese phonological processing (Tan et al., 2005). And the structure and function of the left MFG of Chinese dyslexics were reported abnormal in an fMRI study (Siok et al., 2008) which was consistent with the present result, suggesting phonological processing deficits in Chinese dyslexia. Besides, the LOFC was thought to be a region involved in spatial attention processing (Armony and Dolan, 2002), and this region was often associated with attention deficit in dyslexia (Facoetti et al., 2000). The current results including structural abnormalities in the MFG and LOFC regions were consistent with previous studies suggesting that dyslexics have phonological (Ho et al., 2002; Siok et al., 2008) and attentional (Facoetti et al., 2000) processing deficits. The cerebellar theory also postulates that the cerebellar disorder affecting speech articulation, which would lead to poor phonological representations and phonological skills in dyslexia (Nicolson et al., 2001). As to the relationship between the regions related to phonology/attention and the left cerebellum, the present study can not resolve this, and further research is needed. There is another limitation of the current study, which is the relatively small sample size, so the current findings should be interpreted with caution.

## Conclusion

The present study revealed Chinese children with dyslexia exhibited decreased gray matter volume in the left cerebellum. Moreover, the regional gray matter was significantly correlated with reading scores, suggesting the abnormal structure in the left cerebellum is highly associated with reading disability in Chinese children with dyslexia, supporting cerebellum hypothesis.

## Author Contributions

YY designed and performed the experiments; YY and Y-WZ collected the date; Y-HY performed data analysis and wrote the manuscript. B-GC and H-YB edited the manuscript.

## Conflict of Interest Statement

The authors declare that the research was conducted in the absence of any commercial or financial relationships that could be construed as a potential conflict of interest.

## Footnotes

1 www.fil.ion.ucl.ac.uk/spm

2 http://dbm.neuro.uni-jena.de/vbm.html

3 http://marsbar.sourceforge.net
